# Study the Effect of Heat Treatment on the Corrosion Resistance of AISI 347H Stainless Steel

**DOI:** 10.3390/ma18153486

**Published:** 2025-07-25

**Authors:** Yunyan Peng, Bo Zhao, Jianhua Yang, Fan Bai, Hongchang Qian, Bingxiao Shi, Luntao Wang

**Affiliations:** 1 China Special Equipment Inspection and Research Institute, Beijing 100029, China; pengyunyancsei@163.com (Y.P.); baifan@csei.org.cn (F.B.); 2 State Key Laboratory of Low-Carbon Thermal Power Generation Technology and Equipment, Beijing 102209, China; 3 Institute for Advanced Materials and Technology, University of Science and Technology Beijing, Beijing 100083, China

**Keywords:** AISI 347H stainless steel, solution treatment, corrosion resistance, oxidation resistance, passive film, ToF-SIMS

## Abstract

AISI 347H stainless steel is widely used in high-temperature environments due to its excellent creep strength and oxidation resistance; however, its corrosion performance remains highly sensitive to thermal oxidation, and the effects of thermal history on its passive film stability are not yet fully understood. This study addresses this knowledge gap by systematically investigating the influence of solution treatment on the corrosion and oxidation resistance of AISI 347H stainless steel. The specimens were subjected to solution heat treatment at 1050 °C, followed by air cooling, and then evaluated through electrochemical testing, high-temperature oxidation experiments at 550 °C, and multiscale surface characterization techniques. The solution treatment refined the austenitic microstructure by dissolving coarse Nb-rich precipitates, as confirmed by SEM and EBSD, and improved passive film integrity. The stabilizing effect of Nb also played a critical role in suppressing sensitization, thereby enhancing resistance to intergranular attack. Electrochemical measurements and EIS analysis revealed a lower corrosion current density and higher charge transfer resistance in the treated samples, indicating enhanced passivation behavior. ToF-SIMS depth profiling and oxide thickness analysis confirmed a slower parabolic oxide growth rate and reduced oxidation rate constant in the solution-treated condition. At 550 °C, oxidation was suppressed by the formation of compact, Cr-rich scales with dual-distributed Nb oxides, effectively limiting diffusion pathways and stabilizing the protective layer. These findings demonstrate that solution treatment is an effective strategy to improve the long-term corrosion and oxidation performance of AISI 347H stainless steel in harsh service environments.

## 1. Introduction

AISI 347H stainless steel is a niobium-stabilized austenitic alloy designed for high-temperature service conditions where creep strength, oxidation resistance, and structural stability are required [1,2]. Due to the addition of niobium, AISI 347H exhibits improved resistance to intergranular corrosion by stabilizing chromium carbides in the form of niobium carbides (NbC), thereby preventing chromium depletion along grain boundaries [3,4]. Compared with class-L austenitic stainless steels such as 304L and 316L, which reduce the carbon content to minimize sensitization, AISI 347H relies on the stabilizing effect of Nb to prevent Cr_23_C_6_ precipitation at grain boundaries. This provides improved resistance to intergranular corrosion while maintaining high-temperature strength [5]. This makes it widely applicable in thermal power generation, petrochemical processing, and superheating systems operating at high temperatures [6,7].

Heat treatment has been widely recognized as an effective strategy to enhance the corrosion resistance of stainless steels by modifying microstructure, relieving residual stress, and promoting the formation of stable passive films [8,9,10]. For AISI 347H, heat treatments such as solution treatment and stabilization treatment can significantly influence the distribution of alloying elements [11], the precipitation behavior of niobium carbides, and the overall protective characteristics of the oxide film, especially the Cr_2_O_3_-rich passive layer that plays a crucial role in resisting localized and uniform corrosion [12,13].

Nevertheless, the corrosion resistance of AISI 347H stainless steel remains sensitive to thermal history, particularly in aggressive environments such as chloride-containing aqueous solutions or oxidizing atmospheres [14,15,16]. Therefore, understanding the impact of heat treatment on microstructural evolution and passive film development is essential for improving its long-term performance in service.

Although extensive studies have investigated the corrosion behavior of conventional austenitic stainless steels such as 304 and 316, relatively limited attention has been paid to the effect of heat treatment on the corrosion performance of the high-temperature grade AISI 347H. Given its critical role in energy and petrochemical industries, it is important to clarify how different heat treatment processes influence the corrosion and oxidation resistance of AISI 347H stainless steel under both ambient and elevated temperature conditions.

However, few studies have systematically explored how combined heat treatment affects both the corrosion resistance and high-temperature oxidation behavior of AISI 347H stainless steel. In particular, the underlying mechanisms linking thermal history, residual stress, passive film stability, and oxide scale evolution remain insufficiently understood.

In this study, combined heat treatments, including as-received condition and solution treatment, were applied to AISI 347H stainless steel to systematically evaluate their influence on corrosion and oxidation resistance. The investigation was conducted in two main stages. First, the corrosion resistance of AISI 347H stainless steel with and without heat treatment was assessed using electrochemical techniques and surface characterization methods to understand the impact of thermal processing on passivation behavior. Subsequently, the high-temperature oxidation resistance at 550 °C was examined through detailed surface and compositional analyses using scanning electron microscopy (SEM), X-ray photoelectron spectroscopy (XPS), and time-of-flight secondary ion mass spectrometry (ToF-SIMS). The findings from this work are expected to provide valuable insights into the role of thermal treatment in enhancing the durability of AISI 347H stainless steel under harsh service environments.

## 2. Materials and Methods

### 2.1. Materials and Heat Treatment Methods

The experimental material used in this study was commercial AISI 347H (sourced from Shougang group, Beijing, China) AISI 347H austenitic stainless steel with a nominal chemical composition of 17.13 wt.% Cr, 9.28 wt.% Ni, and 0.53 wt.% Nb. Rectangular specimens with dimensions of 15 mm × 10 mm × 2 mm were sectioned using wire cutting and mechanically ground to remove surface deformation prior to heat treatment.

To investigate the effect of thermal history on microstructure and corrosion performance, two different heat treatment conditions were employed: the as-received condition (347H-AR) and the solution-annealed condition (347H-SA). The solution annealing was performed at 1050 ± 10 °C for 0.5 h, followed by air cooling in ambient atmosphere. This temperature and duration were chosen based on typical industrial practice and literature guidelines for AISI 347H, to ensure sufficient dissolution of precipitates and microstructural homogenization. All heating procedures were conducted in a resistance furnace at a controlled heating rate of 600–900 °C/h, and all specimens were processed in parallel to ensure consistency.

### 2.2. Electrochemical Measurements

Electrochemical measurements were carried out to evaluate the corrosion resistance of AISI 347H stainless steel under different heat treatment conditions. All tests were conducted at room temperature in a conventional three-electrode electrochemical cell using a 3.5 wt.% NaCl aqueous solution as the electrolyte, which simulates a mildly aggressive chloride environment.

The working electrodes were prepared by cutting the samples into 10 mm × 10 mm × 2 mm coupons, which were subsequently ground with SiC papers up to 1200 grit, rinsed with deionized water, degreased with ethanol, and dried under a cold air stream. A platinum sheet served as the counter electrode, while a saturated calomel electrode (SCE) was used as the reference electrode. Unless otherwise specified, all potentials in this study are reported against the SCE.

Prior to each measurement, the working electrode was immersed in the electrolyte for 30 min to establish a stable open circuit potential (OCP). Potentiodynamic polarization tests were performed by scanning the potential from −0.3V to +0.8V relative to the OCP at a scan rate of 1 mV/s. Electrochemical impedance spectroscopy (EIS) was carried out at the OCP over a frequency range of 10^5^ to 10^−2^ Hz with a perturbation amplitude of 10 mV. The obtained impedance spectra were fitted using ZSimpWin software based on an equivalent circuit model to extract electrochemical parameters such as charge transfer resistance (R_ct_) and film resistance (R_f_).

All electrochemical experiments were repeated at least three times to ensure reproducibility, and representative curves are presented in the results section.

### 2.3. High-Temperature Oxidation Tests

High-temperature oxidation experiments were conducted to investigate the oxidation behavior of AISI 347H stainless steel under different heat treatment conditions.

The oxidation temperature of 550 °C was chosen to simulate the typical service environment of AISI 347H stainless steel in high-temperature industrial equipment, where it is commonly exposed to operating conditions between 500 °C and 600 °C. The samples were heated using a furnace ramped up together, ensuring that the heating rate matched the natural temperature rise in the furnace to avoid thermal shock or oxidation artifacts.

For each temperature level, five oxidation durations were set: 2 h, 6 h, 12 h, 24 h, and 48 h. At the end of each oxidation period, the samples were immediately removed from the furnace and air-cooled to room temperature to preserve the high-temperature oxidation products. After cooling, the oxidized samples were stored separately in clean, sealed plastic bags to prevent contamination and further oxidation.

### 2.4. Characterization Methods

The surface morphology and elemental distribution of the oxide layers formed after high-temperature oxidation were examined using a field-emission scanning electron microscope (FE-SEM, JEOL IT-700, Tokyo, Japan) equipped with an energy-dispersive X-ray spectroscopy (EDS) system (Oxford Instruments, Abingdon, UK). Prior to observation, the oxidized samples were ultrasonically cleaned in ethanol and dried in cold air to remove loose surface contaminants.

X-ray diffraction (XRD) analysis was carried out to identify the phase constituents of the samples using a Rigaku SmartLab X-ray diffractometer with Cu Kα radiation (*λ* = 1.5406 Å). The scan was performed in the 2θ range of 10–90° with a step size of 0.02° and a scanning speed of 2°/min. The tube voltage and current were set to 40 kV and 30 mA, respectively. All measurements were conducted at room temperature in the θ–2θ mode.

The corrosion behavior of the alloys was evaluated by a sequence of electrochemical tests including open circuit potential (OCP) stabilization, potentiodynamic polarization, and electrochemical impedance spectroscopy (EIS). Prior to testing, all samples were mechanically ground with SiC papers up to 2000 grit, ultrasonically cleaned in ethanol, and dried in air. The tests were conducted in 3.5 wt.% NaCl solution at room temperature using a conventional three-electrode setup.

The OCP was monitored for 30 min before each measurement. Potentiodynamic polarization curves were recorded in the potential range of −0.3 V to +0.8 V (vs. OCP) at a scan rate of 1 mV/s. EIS measurements were performed at the OCP over a frequency range of 100 kHz to 10 mHz with an amplitude of 10 mV. All electrochemical were obtained by equivalent circuit fitting using ZSimpWin software.

### 2.5. Analysis of Oxidation Products

ToF-SIMS depth profiles were acquired using a ToF-SIMS 5 spectrometer (IONTOF GmbH, Münster, Germany) under a base pressure of 10^−9^ mbar. A pulsed 25 keV Bi^+^ primary ion beam was employed for static analysis, delivering a target current of 1.2 pA over a 100 × 100 μm^2^ area. The beam operated in bunched mode with a pulse width of 1.2 ns. Depth profiling was conducted by alternating static analysis with sputtering, using a 0.5 keV Cs^+^ sputter beam at a current of 17 nA over a 300 × 300 μm^2^ area. Cesium ions (Cs^+^) were chosen for sputtering due to their minimal impact on the secondary ionization yield. Both the Bi^+^ and Cs^+^ ion beams were directed at the sample surface at an incident angle of 45°, and the analyzed ions were collected from the center of the sputtered crater to minimize edge effects. Assuming a constant sputtering rate (0.02 nm·s^−1^) in the oxide, independent of the oxide layer composition, the sputtering time directly translates into oxide thickness.

## 3. Results and Discussion

### 3.1. Microstructure Charact

**Figure 1:**
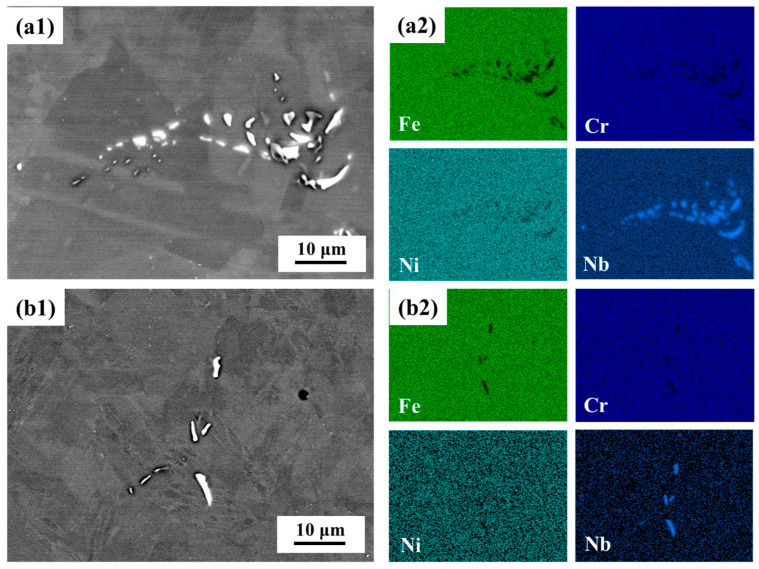
SEM images and corresponding EDS elemental mapping of AISI 347H stainless steel under different heat treatment conditions: (**a1**) 347H-AR, (**a2**) EDS mapping results of 347H-AR, (**b1**) 347H-SA, (**b2**) EDS mapping results of 347H-SA.

Figure 1 illustrates the microstructural characteristics and elemental distributions of AISI 347H stainless steel subjected to two distinct heat treatment processes. In the AR condition (Figure 1a1,a2), a large number of coarse, irregular Nb-rich precipitates are observed, predominantly located at grain boundaries and within the grains. In contrast, the solution-annealed sample (Figure 1b1,b2) exhibits a significantly reduced number of precipitates, with most Nb-rich phases either dissolved or refined into smaller particles. The corresponding EDS elemental maps clearly demonstrate that the bright contrast regions in the SEM images are enriched in Nb, with relatively low Fe and Cr concentrations, indicative of the presence of Nb-type precipitates. The relatively uniform distribution of Fe, Cr, and Ni in the matrix suggests that the heat treatment primarily affects the secondary phase evolution rather than the bulk composition.

**Figure 2:**
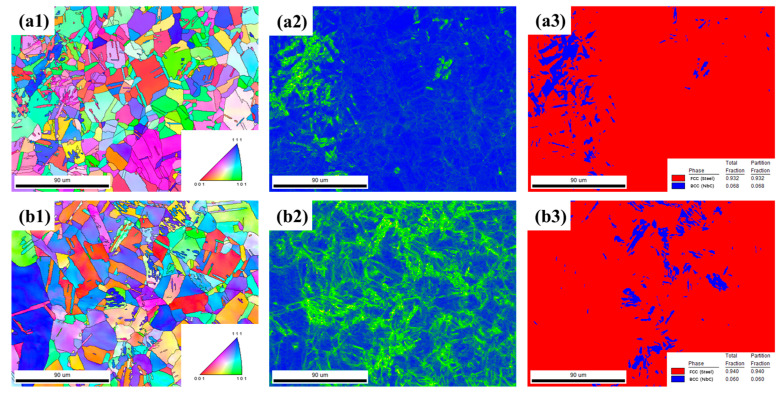
EBSD analysis of AISI 347H stainless steel under different heat treatment conditions: (**a1**–**a3**) AR, (**b1**–**b3**) SA. Each row includes the inverse pole figure (IPF) map, kernel average misorientation (KAM) map, and phase map, respectively.

Figure 2 shows the EBSD characterization results of AISI 347H stainless steels. The inverse pole figure (IPF) maps (a1–b1) reveal that all samples exhibit fully austenitic microstructures with equiaxed grains, while grain refinement is most evident in the solution-annealed condition (b1). The kernel average misorientation (KAM) maps (a2–b2) indicate varying levels of local plastic strain and dislocation density. 347H-SA exhibits localized regions of high KAM intensity, reflecting stress concentration induced by carbide/nitride precipitation.

The phase maps (a3–b3) further confirm that the matrix phase is face-centered cubic (FCC, red), with minor NbC (BCC, blue) phases distributed along grain boundaries and within the matrix. Quantitative phase analysis reveals the ratio of two AISI 347H stainless steels is similar.

**Figure 3:**
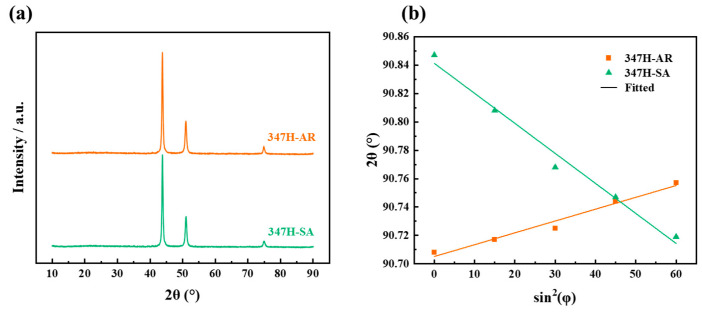
XRD analysis of AISI 347H stainless steel under different heat treatment conditions: (**a**) XRD patterns and (**b**) residual stress analysis based on the sin^2^ψ method using the (311) diffraction plane.

Figure 3a presents the X-ray diffraction (XRD) patterns of AISI 347H stainless steel under two different heat treatment conditions. All samples exhibit typical diffraction peaks corresponding to the austenitic FCC structure, with no detectable transformation to martensitic or BCC phases at the resolution of the instrument.

Figure 3b presents the surface residual stress results obtained via the sin^2^ ψ method based on the (311) diffraction plane, which is widely used for stress analysis in FCC alloys due to its low susceptibility to crystallographic texture and intergranular effects. 347H-SA exhibits the highest residual tensile stress, which may result from thermal mismatch stress and dislocation recovery-induced imbalance during high-temperature annealing. In contrast, the 347H-AR sample demonstrates the lowest residual stress level, reflecting its relatively unperturbed microstructure and lack of significant thermal or phase transformation history. These findings underscore the pronounced influence of thermal treatment on the residual stress state, which could play a crucial role in the subsequent oxidation behavior and mechanical performance.

### 3.2. Electrochemical Properties

**Figure 4:**
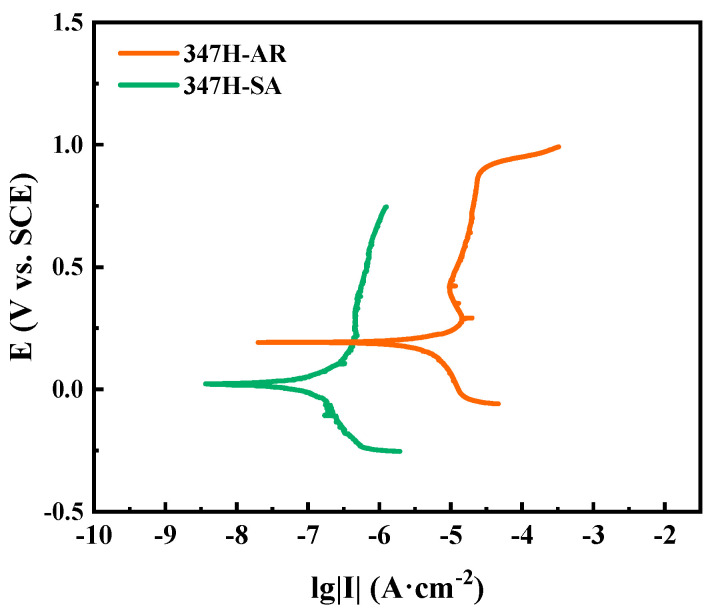
Polarization curve test results of AISI 347H stainless steel under different heat treatment conditions.

Figure 4 presents the potentiodynamic polarization curves of AISI 347H stainless steel subjected to different heat treatment conditions. All samples exhibit characteristic passive behavior with distinct passivation regions, but their corrosion resistance varies significantly depending on thermal history. The 347H-SA sample shows the lowest corrosion current density (I_corr_), indicating its superior ability to form and maintain a stable passive film. This improvement is likely attributed to the optimized distribution of Nb-rich precipitates, which act as Cr-donating sites and enhance passivation. In contrast, the 347H-AR sample exhibits a significantly higher I_corr_, reflecting inferior passivation and greater susceptibility to uniform corrosion. It suggests that while annealing improves microstructural uniformity, the reduction in Nb-containing phases may reduce passivation stability. These findings confirm that stabilization heat treatment plays a beneficial role in improving the corrosion resistance of AISI 347H stainless steel by modifying its precipitate morphology and passive film properties.

**Figure 5:**
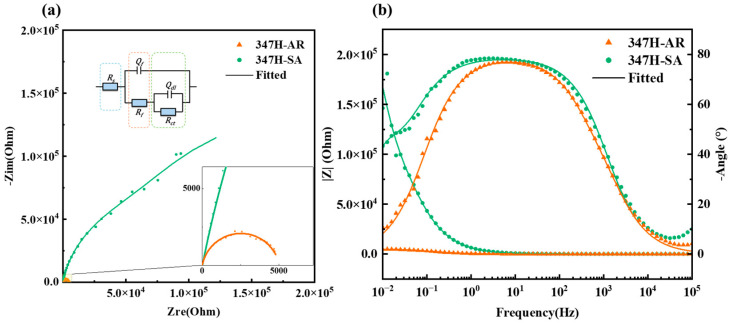
EIS test results of AISI 347H stainless steel under different heat treatment conditions: (**a**) Nyquist plot; (**b**) Bode plot.

The EIS results, shown in Figure 5, further validate the corrosion behavior observed in the polarization measurements. The electrochemical data were fitted using the equivalent circuit model presented in Figure 5a. The Nyquist plots (Figure 5a) exhibit typical depressed capacitive semicircles for all samples, indicating a passive film–electrolyte interface governed by charge transfer processes. The 347H-SA sample exhibits the largest semicircle diameter, corresponding to the highest charge transfer resistance, suggesting a dense and stable passive film. The fitted equivalent circuit model, consisting of two time constants, was used to represent the bilayer structure of the passive film as an outer porous layer and an inner compact layer.

Overall, the EIS analysis confirms that solution treatment significantly enhances the electrochemical stability of the passive film on AISI 347H stainless steel, consistent with its superior polarization performance.

### 3.3. High-Temperature Oxidation Behavior

**Figure 6:**
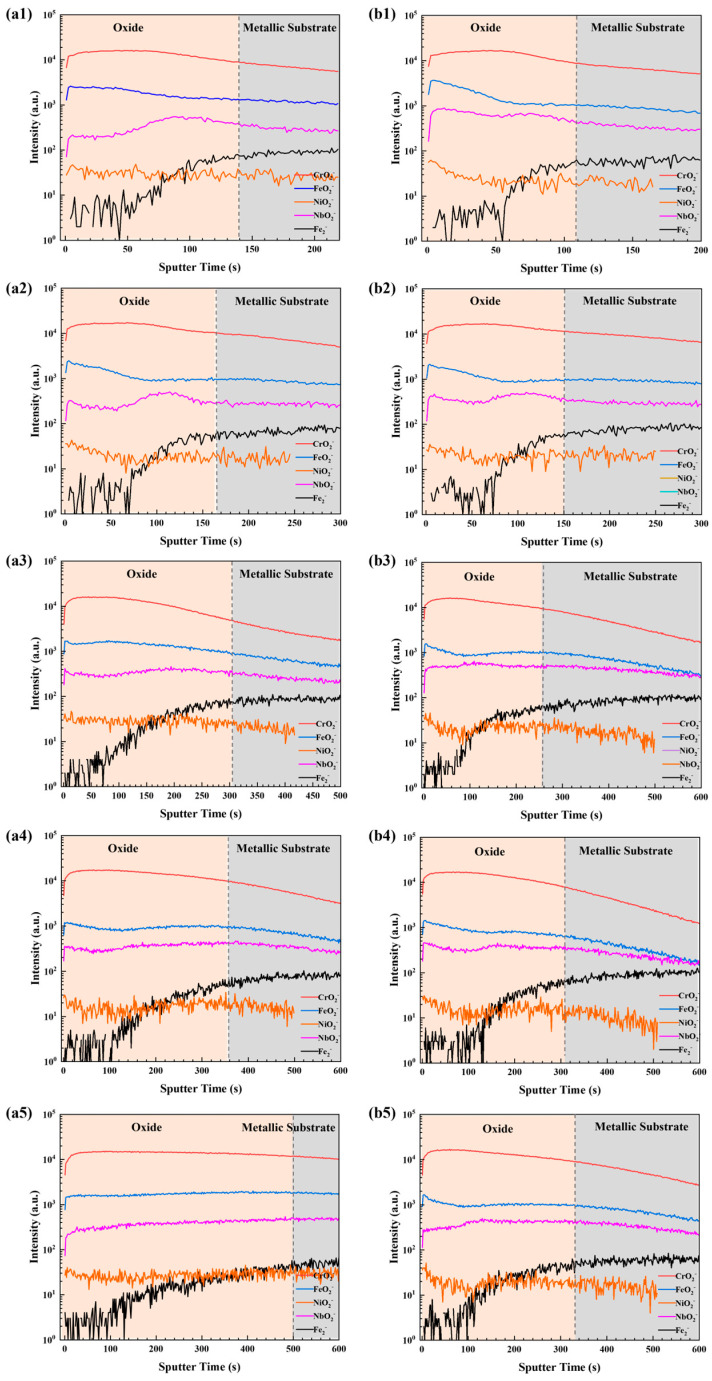
ToF-SIMS depth profiles on the 347H-AR and 347H-SA after oxidation at 550 °C for (**a1**,**b1**) 2 h, (**a2**,**b2**) 6 h, (**a3**,**b3**) 12 h, (**a4**,**b4**) 24 h, and (**a5**,**b5**) 48 h.

The samples were subsequently subjected to oxidation at 550 °C to evaluate the influence of heat treatment on the oxidation resistance of AISI 347H stainless steels. Figure 6 presents the ToF-SIMS negative ion depth profiles of the AISI 347H stainless steel samples with and without heat treatment after different oxidation durations. This technique enabled the identification of specific oxide species as well as their lateral and depth distributions, which complement the morphological and elemental information obtained from SEM and EDS. The signal intensity is plotted on a logarithmic scale as a function of sputtering time. The metallic region is defined by the onset of the intensity plateau of the Fe_2_^−^ ion profile, indicating the interface between the oxide film and the metallic substrate [17].

As shown in Figure 6, FeO_2_^−^, CrO_2_^−^, NiO_2_^−^, and NbO_2_^−^ signals are detected in the oxide region, indicating the presence of iron, chromium, nickel, and niobium oxides, respectively. As shown in Figure 6a1, after 1 h of oxidation of the 347H-AR sample, the FeO_2_^−^ depth profile exhibits a peak at the outermost region and gradually decreases across the entire oxide layer, suggesting that iron oxide is primarily concentrated near the surface, with a smaller fraction distributed within the inner oxide. In contrast, the CrO_2_^−^ signal peaks deeper within the oxide layer and spans a broader region than FeO_2_^−^, indicating that chromium oxide predominantly forms in the inner oxide layer. The significantly higher intensity of the CrO_2_^−^ signal compared to FeO_2_^−^ further suggests that the oxide film is richer in chromium oxide. The NiO_2_^−^ signal also peaks at the surface but exhibits relatively low intensity, implying limited formation of nickel oxide, consistent with previous findings that nickel, due to its lower oxygen affinity, is less prone to oxidation than iron and chromium [18]. Meanwhile, the NbO_2_^−^ signal peaks near the oxide/metal interface, indicating that niobium oxide is mainly concentrated at the inner boundary of the oxide film. Taken together, these profiles suggest that the oxide film formed on 347H-AR after 1 h of oxidation exhibits a bilayer structure, with the outer layer enriched in Fe and Ni oxides and the inner layer dominated by Cr and Nb oxides, where Cr oxide is the principal component.

As for the 347H-SA sample after 2 h of oxidation, the results shown in Figure 6b1 indicate that the oxide film also exhibits a bilayer structure similar to that observed in the 347H-AR sample, with the outer layer enriched in Fe and Ni oxides and the inner layer dominated by Cr and Nb oxides. However, a notable difference is that the NbO_2_^−^ depth profile displays two distinct peaks, one located in the outermost region and the other near the oxide/metal interface, suggesting that Nb oxides are distributed both at the surface and at the substrate interface. This dual distribution of Nb oxides contributes to the superior oxidation resistance of the 347H-SA sample compared to 347H-AR.

Niobium oxides enhance oxidation resistance through multiple mechanisms [19,20]. Due to their intrinsically low ionic conductivity, particularly in the form of Nb_2_O_5_, they serve as effective barriers to oxygen ion diffusion, thereby reducing the inward transport of oxygen and the outward migration of metal cations, which slows down the oxidation process. Furthermore, niobium acts synergistically with chromium to stabilize the Cr_2_O_3_ oxide scale, suppress chromium volatilization and redistribution under high-temperature conditions, and improve the adhesion and resistance to spallation of the protective oxide layer.

With increasing oxidation time, the oxide film structures of both 347H-SA and 347H-AR samples remain unchanged, indicating that prolonged oxidation does not significantly alter the bilayer configuration of the oxide scales on either steel. Regarding the CrO_2_^−^ signal, its intensity remains consistently high throughout the oxidation process, while the FeO_2_^−^ signal gradually decreases. This observation aligns with the fact that chromium has a higher affinity for oxygen than iron, making it more susceptible to oxidation under high-temperature conditions. As for the NbO_2_^−^ and NiO_2_^−^ signals, their intensities remain relatively stable, suggesting that the distribution and oxidation states of niobium and nickel do not significantly vary with extended oxidation time.

**Figure 7:**
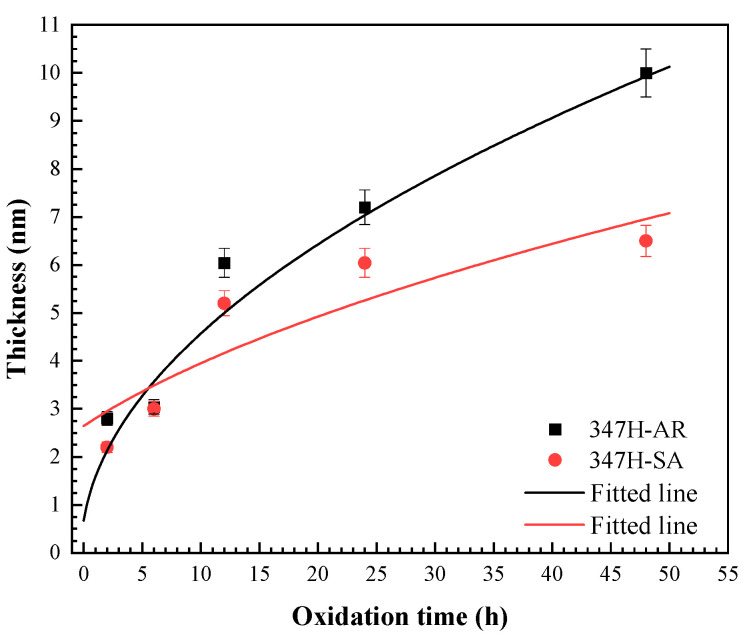
Thickness evolution of 347H-AR and 347H-SA samples exposed at 550 °C as function of exposure time. The curves represent the fitted thickness evolution vs. exposure time curves according to Equation (1).

In Figure 6, the dashed lines mark the boundary between the oxide layer and the underlying metal, allowing for the estimation of oxide thickness based on sputtering time. For the 347H-AR sample, the oxide thickness corresponds to sputtering times of 140 s, 152 s, 302 s, 360 s, and 500 s after oxidation for 2 h, 6 h, 12 h, 24 h, and 48 h, respectively. In contrast, the 347H-SA sample exhibited sputtering times of 110 s, 150 s, 260 s, 302 s, and 325 s for the same oxidation durations. Assuming a sputtering rate of 0.02 nm·s^−1^, these values correspond to oxide thicknesses of 2.8 nm, 3.0 nm, 6.0 nm, 7.2 nm, and 10.0 nm for 347H-AR, and 2.2 nm, 3.0 nm, 5.2 nm, 6.0 nm, and 6.5 nm for 347H-SA, respectively. The complete evolution of oxide thickness is summarized in Figure 7.

As shown in Figure 7, the thickness evolution of AISI 347H stainless steel samples exhibit an approximately parabolic growth behavior throughout the entire exposure duration at 550 °C during. This trend suggests that the oxidation process is controlled by diffusion mechanisms. According to Wagner’s oxidation theory [21], for oxidation processes governed by parabolic kinetics, the relationship between the thickness (nm) and exposure time (t) can be described by the following equation:

(1) $$d^{2}\;=\;k_{p}\cdot t\;+\;C,$$

where d represents the oxide film thickness, t is the exposure time, and *k_p_* is the parabolic rate constant of the oxidation reaction, which is temperature-dependent. According to Equation (1), the parabolic relationship between oxide thickness and exposure time can be fitted using the experimental data obtained at different temperatures, as shown by the dashed curves in Figure 7. Based on the fitting results, the parabolic rate constants at 550 °C were determined to be 2.042 nm^2^/h for the 347H-AR and 0.862 nm^2^/h for the 347H-SA.

These results indicate that the oxide film on 347H-SA grows more slowly than that on 347H-AR under identical oxidation conditions. The reduced oxidation rate of 347H-SA can be attributed to its modified microstructure after heat treatment, which promotes the formation of a more uniform and protective oxide layer with reduced defect density. This enhanced oxidation resistance suggests that appropriate heat treatment can effectively improve the long-term high-temperature performance of AISI 347H SS by stabilizing the passive film and suppressing the outward diffusion of metallic species.

**Figure 8:**
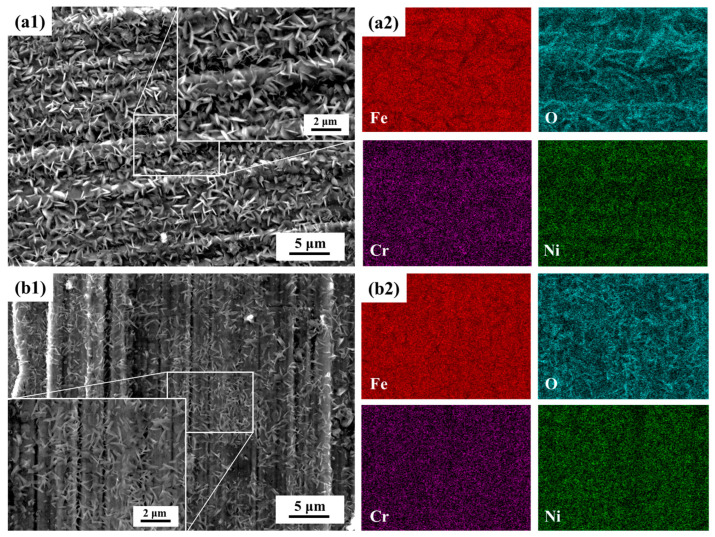
The SEM and EDS analysis on the 347H-AR and 347H-SA samples after oxidation at 550 °C for 48 h, (**a1**) 347H-AR, (**a2**) EDS mapping results of 347H-AR, (**b1**) 347H-SA, (**b2**) EDS mapping results of 347H-SA,.

Figure 8 presents the SEM images and corresponding EDS elemental mapping of 347H-AR and 347H-SA samples after oxidation at 550 °C for 48 h. The surface morphology of the 347H-AR sample (Figure 8a1,a2) reveals dense, randomly oriented needle-like or flake-like oxide structures, while the 347H-SA sample (Figure 8b1,b2) exhibits more aligned and uniform oxide features, suggesting a more compact and ordered oxide scale. The higher degree of structural regularity in the 347H-SA sample indicates improved surface protection under prolonged oxidation. EDS mapping confirms the presence of Fe, Cr, Ni, and O in the oxide films of both samples. In both cases, strong Cr and O signals suggest that the oxide scales are primarily composed of Cr-rich oxides, most likely Cr_2_O_3_. Fe is also distributed across the surface, indicating partial formation of iron oxides, while Ni appears with relatively weaker intensity. Notably, the 347H-SA sample shows a more homogeneous and dense distribution of Cr and O, supporting its superior oxidation resistance compared to the 347H-AR sample.

Similar corrosion behaviors were observed by Chen et al. [2] in their study of TP347H stainless steel exposed to supercritical water. At 550 °C, they also reported parabolic oxidation kinetics and the formation of bilayer oxide scales consisting of outer Fe-based oxides and inner (Fe,Cr)-rich spinels. In our work, although the oxidation environment differs (dry air vs. supercritical water), the solution-treated AISI 347H steel similarly developed a compact, Cr_2_O_3_-rich inner layer that limited oxygen diffusion and stabilized the oxide structure.

## 4. Conclusions

Solution treatment significantly refines the microstructure of AISI 347H stainless steel by dissolving or reducing coarse Nb-rich precipitates and promoting a uniform austenitic matrix. EBSD and XRD analyses confirm grain refinement, phase homogenization, and a higher residual tensile stress state, which may influence oxidation behavior. These microstructural changes improve the overall material uniformity and reduce potential corrosion initiation sites.

Electrochemical testing shows that the solution-treated sample exhibits lower corrosion current density and higher charge transfer resistance, indicating enhanced passivation behavior. The EIS results further reveal the formation of a more stable bilayer passive film. These findings demonstrate that solution treatment improves the corrosion resistance of AISI 347H stainless steel by optimizing precipitate distribution and passive film integrity, offering better durability in aggressive environments.

Solution treatment significantly improves the high-temperature oxidation resistance of AISI 347H stainless steel at 550 °C. ToF-SIMS depth profiling revealed that both the as-received and solution-annealed samples form bilayer oxide films, with outer layers rich in Fe/Ni oxides and inner layers dominated by Cr/Nb oxides. However, the 347H-SA sample exhibited a distinct dual distribution of Nb oxides—at both the surface and the oxide/metal interface—suggesting a stronger barrier effect. The refined microstructure and redistribution of Nb contributed to the formation of a more protective oxide layer, as further supported by slower oxide growth kinetics.

Oxide thickness measurements confirmed that the 347H-SA sample experienced significantly slower parabolic growth, with a lower oxidation rate constant (0.862 nm^2^/h) than the 347H-AR (2.042 nm^2^/h), indicating enhanced resistance to oxygen diffusion. SEM/EDS analyses after 48 h of oxidation further showed that the SA-treated sample formed a more compact and uniform Cr-rich oxide scale, while the AR sample exhibited irregular and porous oxide morphologies. These results demonstrate that solution treatment enhances long-term oxidation resistance by stabilizing Cr_2_O_3_ scales, suppressing defect formation, and reducing the diffusion of reactive species under high-temperature conditions.

Overall, this study provides a mechanistic understanding of how solution treatment affects the corrosion and oxidation behavior of AISI 347H stainless steel at service-relevant temperatures, addressing the knowledge gap in linking thermal history to passive film stability and high-temperature durability.

## Data Availability

The raw data supporting the conclusions of this article will be made available by the authors on request.
